# Quantitative Evaluation of Gait Disturbance on an Instrumented Timed Up-and-go Test

**DOI:** 10.14336/AD.2018.0426

**Published:** 2019-02-01

**Authors:** Shigeki Yamada, Yukihiko Aoyagi, Kazuo Yamamoto, Masatsune Ishikawa

**Affiliations:** ^1^Normal Pressure Hydrocephalus Center, Rakuwakai Otowa Hospital, Rakuwakai Healthcare System, Kyoto, Japan.; ^2^Department of Neurosurgery and Stroke Center, Rakuwakai Otowa Hospital, Rakuwakai Healthcare System, Kyoto, Japan.; ^3^Digital Standard Co., Ltd., Osaka, Japan.; ^4^Rakuwa Villa Ilios, Rakuwakai Healthcare System, Kyoto, Japan

**Keywords:** gait disturbance, gait assessment, diagnostic test assessment, timed up-and-go test, idiopathic normal-pressure hydrocephalus, cerebrospinal fluid tap test

## Abstract

Although the 3-m timed up-and-go test (TUG) is reliable for evaluating mobility, TUG time is insufficient to evaluate mild gait disturbance; we, therefore aimed to investigate other measurements with instrumented TUG (iTUG) using a free smartphone application. Our inclusion criterion in this study is only that participants can walk without any assistance. This study included three heterogeneous groups; patients who underwent a tap test or shunt surgery, 29 inpatients hospitalized for other reasons, and 87 day-care users. After the tap test, 28 were diagnosed with tap-positive idiopathic normal-pressure hydrocephalus (iNPH) and 8 were diagnosed with tap-negative. Additionally, 18 patients were assessed iTUG before and after shunt surgery. During iTUG, time and 3-dimensional (3D) acceleration were automatically recorded every 0.01 s. A volume of the 95% confidence ellipsoid (95%CE) of all plots for 3D acceleration was calculated. Additionally, an iTUG score was defined as (95%CE volume) ^0.8^ / 1.9 - 1.9 × (time) + 60. The measurement reliability was evaluated using intraclass correlations and Bland-Altman plots. The participants with mild gait disturbance who accomplished within 13.5 s on the iTUG time had the 95%CE volumes for 3D acceleration of ≥70 m^3^/s^6^ and iTUG scores of ≥50. The mean iTUG time was shortened and the mean 95%CE volumes and iTUG scores were increased after the tap test among 28 patients with tap-positive iNPH and after shunt surgery among 18 patients with definite iNPH. Conversely, the mean iTUG score among 8 patients with tap-negative was decreased after the tap test. The intraclass correlations for the time, 95%CE volume and iTUG score were 0.97, 0.80 and 0.90, respectively. Not only the iTUG time but also the 95%CE volume was important for evaluating mobility. Therefore, the novel iTUG score consisting both is useful for the quantitative assessment of mobility.

The 3-m timed up-and-go test (TUG) is widely used as a quantitative measure for assessing general mobility in healthy elderly and patients with various diseases [1-8]. The TUG is also used for quantitatively evaluating the improvement in gait disturbance after a shunt surgery or tap test involving 30-50 mL removal of cerebrospinal fluid (CSF) via a lumbar tap in patients with idiopathic normal-pressure hydrocephalus (iNPH)[9-13]. Recently, the time on TUG at the tap test was proposed to be a reliable quantitative measure for predicting gait improvement after shunt surgery [13]. For patients with iNPH whose TUG time shortened by ≥5 s after the tap test, there is an almost 40% expectation of ≥10s improvement on TUG time at 12 months after shunt surgery and an almost 65% expectation of ≥5s improvement. Conversely, for patients whose TUG time did not shorten or shortened by <5s after the tap test, diligent decision-making is needed. However, the TUG time is not adequate for evaluating patients with mild gait disturbance. For example, a TUG time of 11 s before the tap test could not be reduced by ≥5s after the tap test or shunt surgery. Therefore, in this study, we investigated the key element other than time for evaluating mild gait disturbance using an instrumented TUG (iTUG) with a free iPhone application. Furthermore, we established a novel universal score for assessing severity of gait disturbance and mobility in healthy elderly and patients with various diseases, taking the place of the time on TUG.

**Figure 1.**
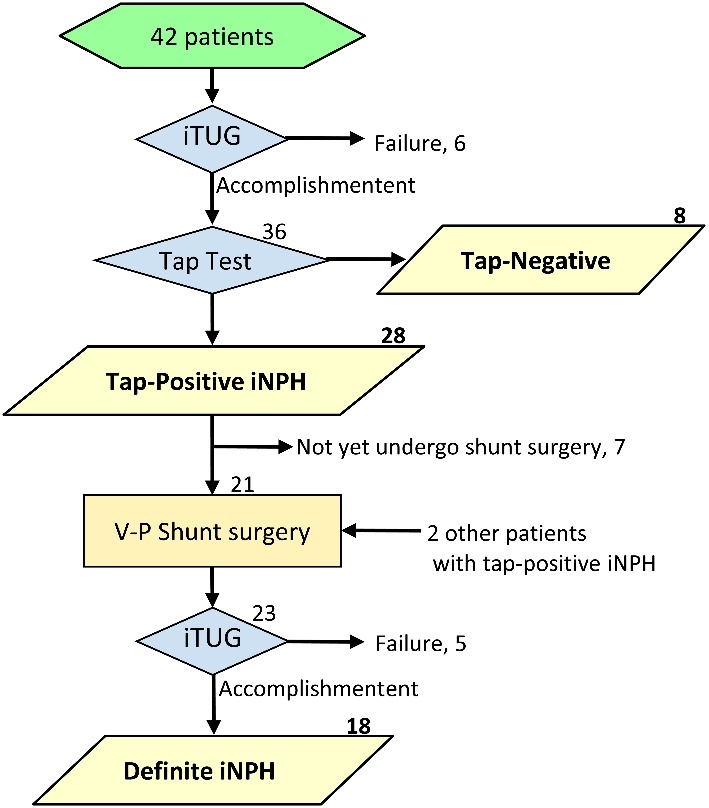
**Flow chart for patient selection in this study**. Yellow parallelograms indicate the patients included in the study.

## MATERIALS AND METHODS

### Study population

The study design and protocol were approved by the ethics committee for human research at our two institutes. Our inclusion criterion in this study is only that participants can walk without any assistance. We asked for the participation after explanation of the study purpose and design at our hospital and rehabilitation facility. The participants were recruited, and their private information was anonymized in a linkable manner at each institute since March 2017. After the participants or their representatives provided written informed consent, they underwent iTUG and a 15-step walk test, two times for each test.

A total of 42 consecutive patients underwent the CSF tap test, which consisted of removing 30 to 40 mL CSF via a lumbar tap to assess for the presence of iNPH and to predict the response to shunt surgery. As shown in the flow chart (Fig. 1), 6 patients (14%) could not perform iTUG before the tap test, because of time-consuming for standing or start walking, namely magnetic gait. Therefore, 36 patients who accomplished iTUG were included in this study. Response to the tap test was assessed by the iNPH grading scale and the quantitative examination of gait and cognition before, 1 day and 4 days after the CSF tap test, according to the Japanese iNPH guidelines [14]. On the basis of the response to the tap test, 28 patients were diagnosed as having “tap-positive iNPH” and the other 8 patients were diagnosed with “tap-negative”. Of the 28 patients with tap-positive iNPH, 21 patients underwent ventriculoperitoneal shunt surgery from 2 weeks to 8.5 months (median 1.8 months) after the tap test, whereas 7 patients had not yet for the following reasons: 4 patients are planned to undergo shunt surgery within 2 months, 2 patients had a mild gait disturbance and lasting improvement of their gait disturbance after the tap test, and 1 patient had a high perioperative risk and had fear of surgery. Of the 21 patients who underwent shunt surgery, 5 patients with severe gait disturbance could not perform iTUG before the shunt surgery due to the progression of their gait disturbance or surgery continuously after the tap test. Additionally, 2 patients who could be assessed only before and after shunt surgery were included in this study, because they underwent the tap test before the study start. In total, we assessed iTUG data in 28 patients with tap-positive iNPH, 8 patients with tap-negative before and after the tap test, and 18 patients with definite iNPH before and after the shunt surgery. Definite iNPH was diagnosed based on improvement of at least one symptom for iNPH after shunt surgery, in accordance with the Japanese guideline for management of iNPH. To assess various severities of gait in healthy aged persons and patients with various diseases, we recruited another two heterogeneous groups; 29 patients hospitalized for various diseases who did not undergo the tap test at our hospital and 87 trainees who exercised at our rehabilitation facility as day-care users were also recruited. The main reasons for hospitalization in the 29 patients were cancers (8), lumbar canal stenosis (4), cardiac heart disease (3), multiple lumbar compression fracture (2), pneumonia (2), dementia (2), gonarthrosis (2), head injury (2), cervical disc herniation (1), ileus (1), Wernicke’s encephalopathy (1), and Parkinson disease (1). Four patients had a medical history of cerebral infarction. All 29 patients needed the physical rehabilitation mainly for the disuse muscle weakness. All of 87 trainees were in good health condition and did not need the hospitalization at the participation in this study.

### Quantitative measurements

The iPhone application SENIOR Quality (Digital Standard Co., Ltd., Osaka, Japan) is freely downloadable from the Apple store (https://itunes.apple.com/jp/app/seniorquality/id1081764213?mt=8). As shown in Movie 1 (https://youtu.be/0TCgxw1FTo0), the participants were placed the iPhone into a small pouch upon the umbilicus, and then they prepared to start the test while sitting on the chair and setting the iTUG in the SENIOR Quality application. Upon hearing the voice signal (directing to start) from the iPhone, the participants stood up and walked a distance of 3 m, turned around a small cone, returned back to the chair, turned 180°, and sat down as quickly as possible. As the next examination with the SENIOR Quality application, the 15-step walk test was simultaneously conducted with a 10-m straight walk test. In the 15-step walk/10-m straight walk tests, the participants prepared to start in the standing position and started to walk straight until beyond the line of 10 m. The application automatically stopped recording when the participants reached the 15th step.

**Figure 2.**
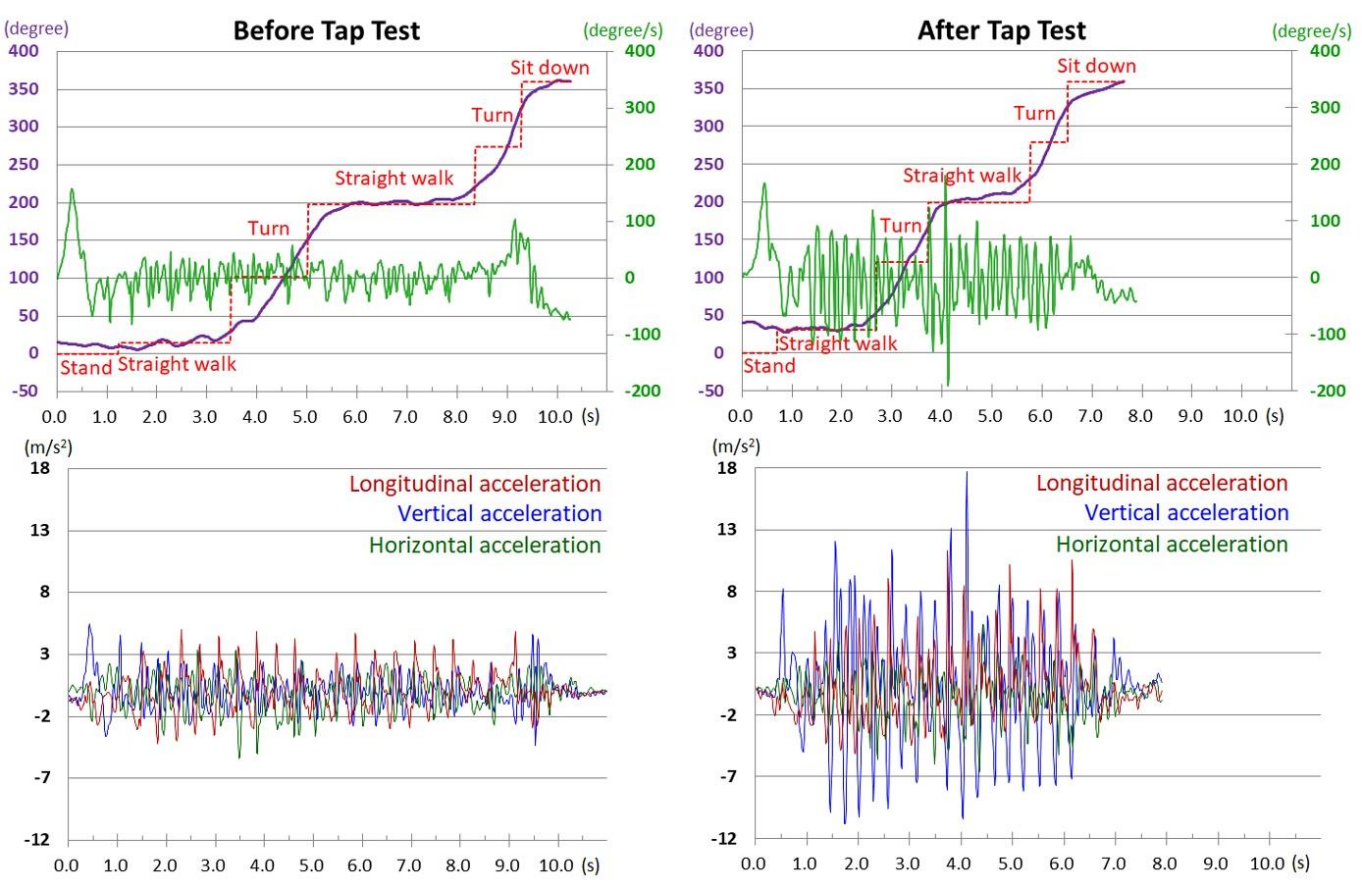
**Motion analysis in three axial directions during instrumented 3-m timed up-and-go test (iTUG)**. A 75-year-old woman underwent the tap test to assess the diagnosis of iNPH. The time on iTUG before the tap test (left) was 10.25 s and shortened 7.88 s after the tap test (right). On the basis of the turn angle (degree, purple) and angular speed for bob movement (degree/s, light green) measured every 0.01 s by an inertial gyroscope, movement during the iTUG was automatically segmented into stand up, go straight forward, turn, go back, turn, and sit down (upper panel). During the iTUG, acceleration in three axial directions was also automatically recorded every 0.01 s (lower panel) by an acceleration sensor. Longitudinal acceleration (red) indicates acceleration toward forward (+) and backward (-), vertical acceleration (blue) indicates acceleration toward upward (+) and downward (-), and horizontal acceleration (green) indicates acceleration toward left (+) and right (-).

### Data acquisition and analysis

By using an inertial gyroscope and accelerometer in the iPhone, angular speed, position coordinate, Euler angle, and acceleration in three axial directions were automatically recorded every 0.01 s and automatically stored in the cloud server (Microsoft Azure; Microsoft Corporation, Redmond, WA, USA). By the angular speeds of the iPhone, movements during the iTUG were automatically segmented into stand up, go forward, turn, go back, turn, and sit down (Fig. 2, upper panel). During the iTUG, the longitudinal (forward and backward), horizontal and vertical acceleration cyclically moved in each step (Fig. 2, lower panel). The forward acceleration indicates the force to advance in the forward direction which is generated by kicking out with a toe. Contrary, the backward acceleration generated by landing the heel is also absolutely necessary for a walk. Additionally, the left-and-right horizontal acceleration which is the force to move the center of gravity to one foot at the beginning walk and the vertical upward acceleration generated by kicking out with a toe is also very important for an ideal gait.

**Figure 3.**
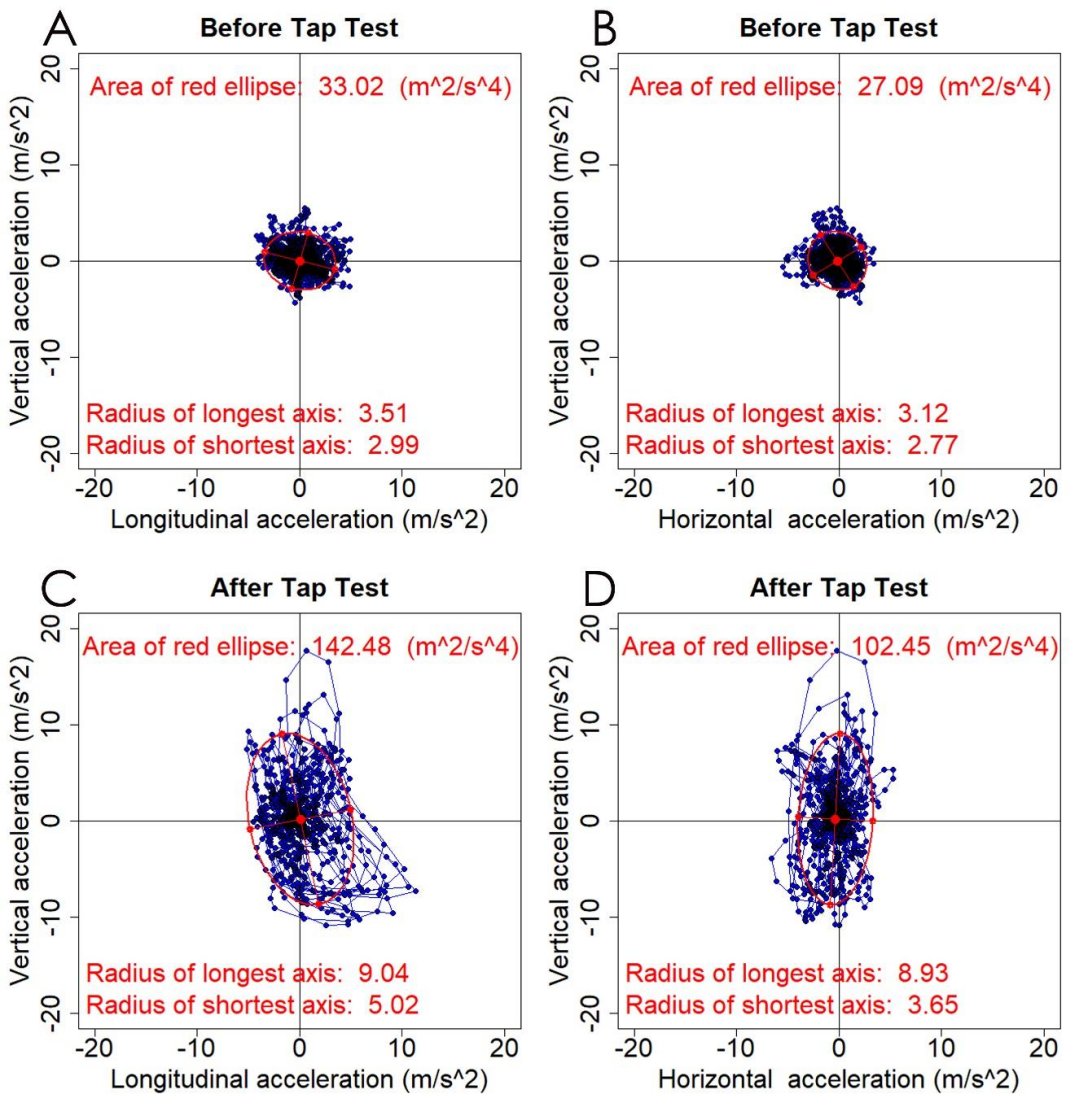
**Two-dimensional (2D) scatter plots and their 95% confidence ellipse for 2D acceleration**. Blue plots indicate the changes of 3D acceleration every 0.01 s in the same case as shown in Figure 2. The red ellipse indicates a 95% confidence ellipse that contains 95% of all plots. Before the tap test, the chronological changes of the plots both in the longitudinal and vertical acceleration (A) and horizontal and vertical acceleration (B) were very small, and the areas of the red ellipses were calculated as 33.0 m^2^/s^4^ and 27.1 m^2^/s^4^, respectively. After the tap test, not only forward acceleration but also upward and downward vertical acceleration was increased. The area of the red ellipse for the longitudinal and vertical acceleration was calculated as 142.5 m^2^/s^4^ (C), and that for the horizontal and vertical acceleration was102.5 m^2^/s^4^ (D).

**Figure 4.**
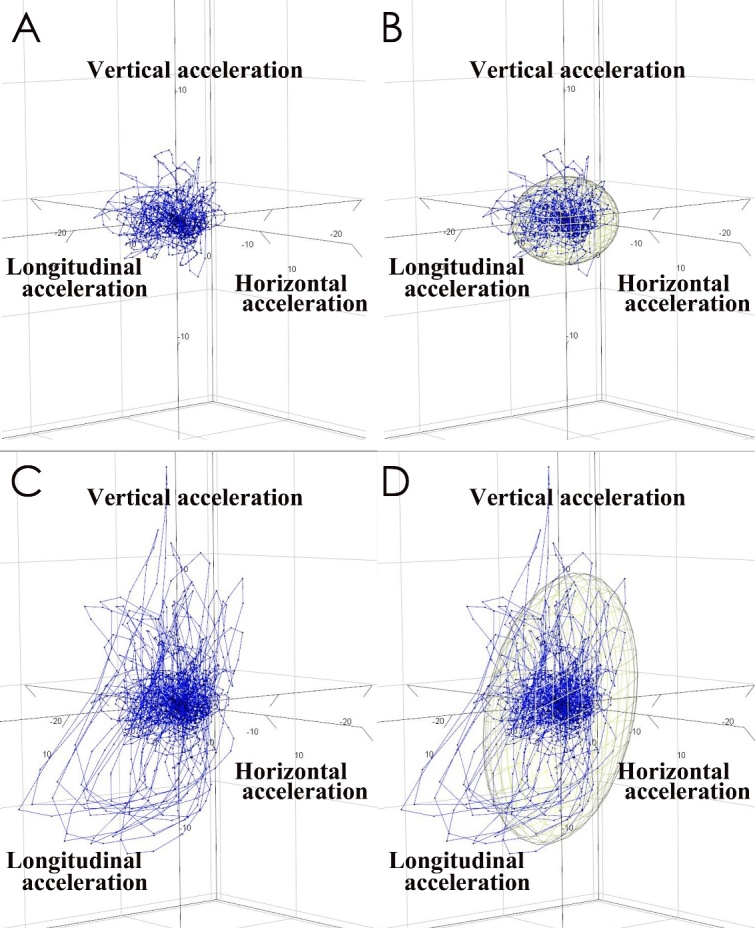
**Three-dimensional (3D) plots and their 95% confidence ellipsoid (95%CE) for 3D acceleration**. Blue plots indicate acceleration every 0.01 s in the same case as shown in Figure 2 and 3. Accelerations were very small in all three axial directions before the tap test (A and B), but they changed larger after the tap test (C and D). The yellow ellipsoids (B and D) indicate a 95% CE that contains 95% of all plots in the 3D graph. The 95%CE volume before the tap test was calculated as 95.19 m^3^/s^6^ (B) and increased to 552.10 m^3^/s^6^ after the tap test (D).

At first, we drew the chronological changes of acceleration in the longitudinal and vertical directions on the two-dimensional (2D) scatter plots and their 95% confidence ellipse (Fig. 3, left panel) using the car package in R (version 3.3.2; R Foundation for Statistical Computing, Vienna, Austria; http://www.R-project.org). Second, 2D scatter plots and their 95% confidence ellipses of horizontal and vertical acceleration were assessed (Fig. 3, right panel). Among the area, length and ratio of major and minor axes of the 95% confidence ellipses of the 2D acceleration on iTUG, we found that the area was the most important for gait assessment. Furthermore, we recognized that all three directions of acceleration had closely related each other and equally important for gait assessment. Therefore, we finally assessed the chronological changes of acceleration in three axial directions on the 3D scatter plots and their 95% confidence ellipsoid (95%CE), as shown in Figure 4 and Movie 2 (https://youtu.be/s85w_TDt1WI). The volume of the 95%CE was calculated as 4π/3 × (maximum length of the axis from the center to the ellipse) × (minimum length) × (length of the axis orthogonal to the two axes). As a result, we found that not only the iTUG time but also the volume of the 95%CE for the 3D acceleration on iTUG were very important for evaluating gait disturbance. Therefore, we newly created the iTUG score, which simultaneously reflects both the iTUG time and the volume of the 95%CE for the 3D acceleration on iTUG, as calculated using the following formula: (95%CE volume) ^0.8^ / 1.9 - 1.9 × (time) + 60. The exponential value of 0.8 and the slope of 1.9 were set to exhibit an almost normal distribution in the range of 0-100 points, and the Y-intercept was set to 60 so that the median of the iTUG score would be 50 points. In this iTUG score, a score of 100 or more indicates good mobility, a score of 50 indicates mild disability to move which corresponds to an iTUG time of 13.5 s and 95%CE volume of 70 m^3^/s^6^, and zero or less indicates inability to walk.

**Table 1:** Baseline characteristics before tap test or shunt surgery.

	Tap +	Tap -	*P* value	Shunt
Total number	28	8		18
Male: Female	18: 10	2: 6	0.12	12: 6
History of falls				
none: 1 or 2 times: ≥3 times	8: 6: 14	2: 1: 5	0.79	7: 4: 7
Co-morbidity				
Alzheimer’s disease	11 (39%)	3 (38%)	1.00	4 (22%)
spinal disease	6 (21%)	4 (50%)	0.25	5 (28%)
cerebral infarction	1 (4%)	0	1.00	2 (11%)
Age, year	77.5 ± 5.9	78.3 ± 9.0	0.82	76.6 ± 5.1
Disease duration, month	33.5 ± 24.0	35.0 ± 10.2	0.29	34.8 ± 21.0
Duration time to shunt, day	-	-		75.1 ± 61.3
	Before tap	Before tap		Before shunt
modified Rankin scale, point	2.6 ± 0.9	2.6 ± 0.7	0.90	2.4 ± 0.6
3-m timed up-and-go test, s	24.9 ± 20.3	16.3 ± 7.0	0.39	14.6 ± 5.8
10-m straight walk test, s	16.0 ± 17.3	12.7 ± 5.1	0.97	11.2 ± 3.8
MMSE, point	22.5 ± 6.9	23.5 ± 3.3	0.82	24.1 ± 4.5
FAB, point	10.5 ± 3.3	10.5 ± 2.3	0.92	12.4 ± 4.4

Tap +; patients diagnosed with tap-positive iNPH Tap -; patients diagnosed with tap-negative *P*; probability value of Tap + vs. Tap - Shunt; patients with iNPH who underwent ventriculoperitoneal shunt surgery Disease duration; time interval from the initial presentation of the symptoms to the tap test Duration time to shunt; time interval from the tap test to the shunt surgery

### Statistical analysis

To evaluate the reliability of the measurements on iTUG, two times of iTUG were conducted, and the intraclass correlation (ICC) and 95% CIs for absolute test-retest reliability were calculated. According to the coefficients of ICC, measurements were interpreted as having excellent reliability for an ICC of ≥0.9, good reliability for 0.8 ≤ ICC < 0.9, acceptable reliability for 0.7 ≤ ICC < 0.8, questionable reliability for 0.6 ≤ ICC <0.7, and poor reliability for an ICC of <0.6. The test-retest agreements were also examined using the Bland-Altman method. We drew the Bland-Altman plots and calculated the mean differences and 95% confidential intervals (CIs) for the two times of the iTUG time, volume of the 95%CE for the 3D acceleration and iTUG score, respectively. Moreover, mean values and SDs for age, disease duration of time from the initial presentation of their symptoms to the tap test and several parameters of iTUG, etc. were calculated and compared using a Mann-Whitney-Wilcoxon test. The ratio of sex, history of falls and the presence of co-morbidities were examined using Pearson’s correlation coefficient (*r*) and 95% CIs. Statistical significance was assumed at a probability value (*p*) of <0.05. Missing data were treated as deficit data that did not affect other variables. All statistical analyses were performed using the R software.

**Table 2:** Clinical characteristics in the groups with <13.5 and ≥13.5 seconds (s) on iTUG time before the tap test.

Initial TUG time	<13.5 s	≥13.5 s	*P* value
Total number	10	18	
Male: Female	8: 2	10: 8	0.38
History of falls			
none: 1 or 2 times: ≥3 times	4: 3: 3	4: 3: 11	0.29
Co-morbidity			
Alzheimer’s disease	2 (20%)	9 (50%)	0.25
spinal disease	2 (20%)	4 (22%)	1.00
cerebral infarction	0	1 (6%)	1.00
Age, year	73.5 ± 5.2	79.8 ± 5.2	<0.01
Disease duration, month	32.9 ± 28.9	33.9 ± 21.8	0.63
modified Rankin scale, point	2 ± 0	3 ± 0.9	<0.01
MMSE, point	26.0 ± 2.4	22.8 ± 5.9	0.06
FAB, point	11.8 ± 3.4	11.9 ± 5.6	0.06
10-m straight walk test, second	8.5 ± 1.3	12.9 ± 3.0	<0.01

*P*; probability value of the group with <13.5 vs. ≥13.5s on iTUG time before the tap test

## RESULTS

### Clinical characteristics

There were no significant differences between the tap-positive iNPH and tap-negative (Table 1) groups in the mean values of age, disease duration, initial times on the TUG and 10-m straight walk test and initial scores of MMSE and FAB or frequency of co-morbidities. More than 70% of patients who underwent the tap test had a history of falls. As shown in Table 2, of 18 patients with tap-positive iNPH whose initial TUG time was ≥13.5s, 11 (61%) had a history of ≥3-times falls, which was two times higher proportion than the patients with <13.5s on initial TUG time (30%). There was no statistical difference in the disease duration from the initial presentation to the tap test between the tap-positive iNPH patients with an initial iTUG time of ≥13.5s (mean ± SD, 31.7 ± 29.0 months) and those with <13.5s (34.6 ± 21.7 months).

### Relationships among time, 95%CE volume, and score on iTUG

**Figure 5.**
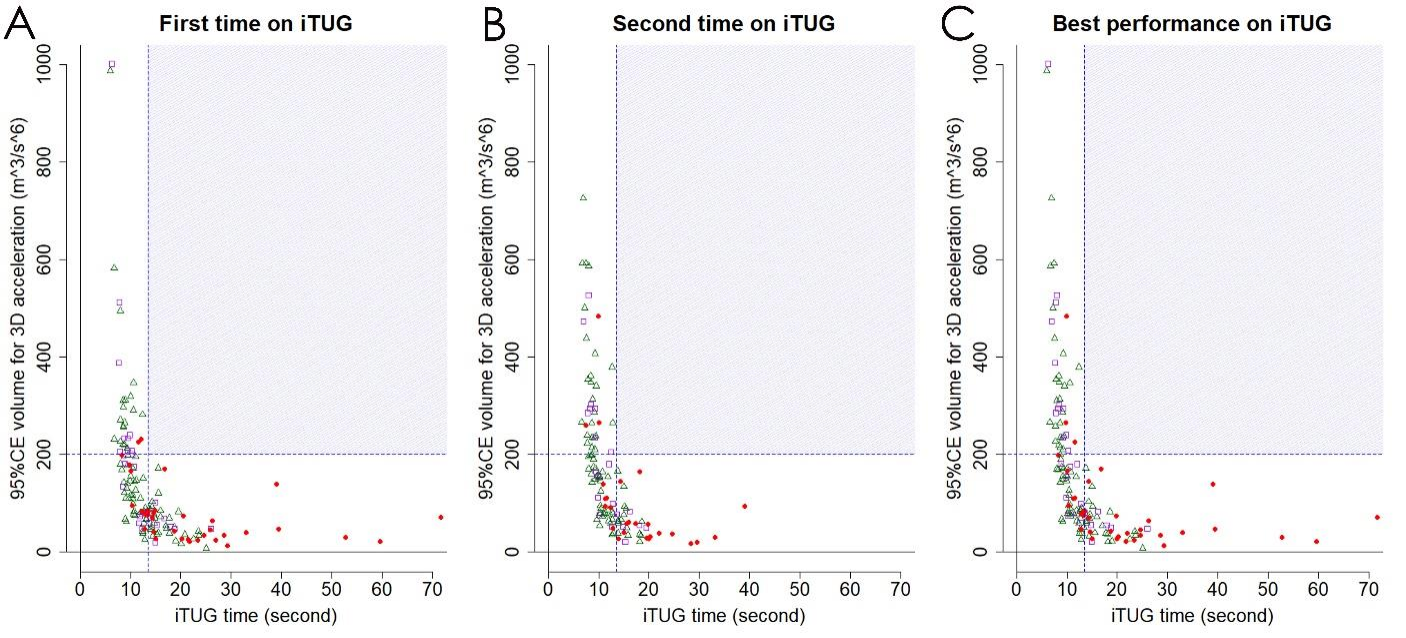
**Scatter plots for time and volume of 95% confidence ellipsoid (95%CE) for 3D acceleration**. The x-axis shows the time on iTUG, and the y-axis shows the 95%CE volume at the first time (A), the second time (B), and the best performance in the two times of iTUG (C). Red circles indicate patients with tap-positive iNPH before the tap test. Purple open squares indicate patients hospitalized for other reasons and did not undergo the tap test, and green open triangles indicate day-care users. There are inverse relationships between the time and 95%CE volume for the 3D acceleration.

**Figure 6.**
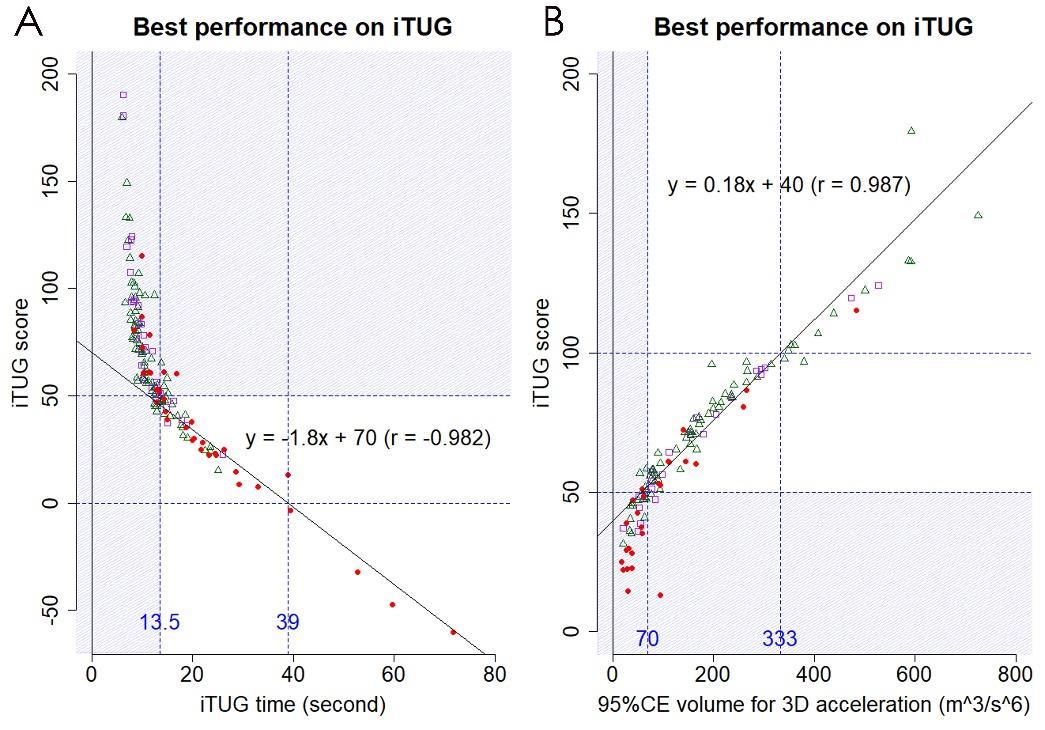
**Scatter plots for the correlations among time, volume of 95% confidence ellipsoid (95%CE) for 3D acceleration and iTUG score**. The left panel (A) indicates the relationships between the time on iTUG (x-axis) and iTUG score (y-axis) at the best performance in the two times on iTUG. The time on iTUG had a negative correlation to the iTUG score in a linear fashion (y = -1.8x + 70) at the iTUG time of >13.5 s and iTUG scores of <50. The right panel (B) indicates the relationships between the 95%CE volume for the 3D acceleration (x-axis) and iTUG score (y-axis). The 95%CE volume had a positive correlation to the iTUG score in a linear fashion (y = 0.18x +40) at the 95%CE volume of ≥70 m^3^/s^6^ and the iTUG scores of ≥50. Red circles indicate patients with tap-positive iNPH before the tap test. Purple open squares indicate patients hospitalized for other reasons, and green open triangles indicate day-care users.

The scatter plots shown in Figure 5 demonstrate the inverse relationship between time and 95%CE volume for the 3D acceleration on iTUG among 144 participants in the three heterogeneous groups. None of the patients had an iTUG time of >13.5 s and 95%CE volume of >200 m^3^/s^6^. The times on iTUG in the 28 patients with tap-positive iNPH tended to longer than those in the 29 patients hospitalized for other reasons and 87 day-care users, whereas the 95%CE volumes for the 3D acceleration in the tap-positive iNPH group tended to smaller than those in the other two groups (Fig. 5). Table 3 shows the measurements on iTUG in the 29 patients hospitalized for other reasons and 87 day-care users. Although there were no significant differences between the two groups in all of the measurements on iTUG, the mean time on iTUG in the 29 inpatients was faster than that in the 87 day-care users at the first time of iTUG. In contrast, the mean values of 95%CE volume and iTUG score at the first time in the 29 inpatients were higher than those in the 87 day-care users. Figure 6 shows the relationships among time, 95%CE volume and score on iTUG. At iTUG scores <50, which were almost equal to an iTUG time of >13.5 s and 95%CE volumes of <70 m^3^/s^6^, the iTUG scores were significantly associated with the iTUG time in a complete linear fashion (*r* = -0.982, linear regression model: y = -1.8x + 70). In contrast, at ≥50 iTUG scores, which were almost equal to an iTUG time of ≤13.5 s and 95%CE volumes of ≥70 m^3^/s^6^, the iTUG scores were significantly associated with the 95%CE volumes in a complete linear fashion (*r* = 0.987, linear regression model: y = 0.18x +40). The patients whose iTUG scores were zero or less had a severe disability to move, which were almost equal to an iTUG time of >39 s, according to the model: y = -1.8x + 70. Conversely, the participants with the iTUG scores ≥100, which were almost equal to a 95%CE volume of ≥333 m^3^/s^6^ according to the model: y = 0.18x + 40, were assessed as a good mobility or normal gait.

### Changes in time, 95%CE volume, and score on iTUG after tap test or shunt surgery

**Figure 7.**
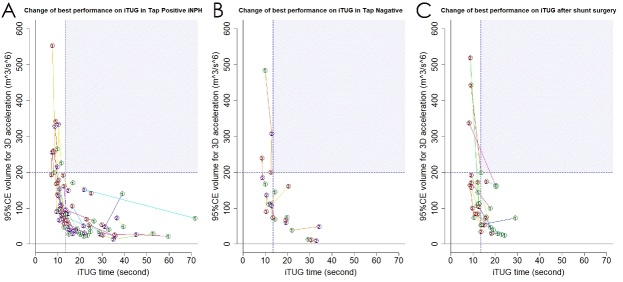
**Changes of the best performance at tap test and shunt surgery**. The time and volume of 95% confidence ellipsoid (95%CE) for 3D acceleration on iTUG before the tap test (number 1, green marks) move to the number 2 (purple marks) 1 day after the tap test, and move to the number 3 (brown marks) 4 days after the tap test among 28 patients with tap-positive iNPH (A) and 8 patients with tap-negative (B). In the same way, those before the shunt surgery (number 1, green marks) move to the number 2 (brown marks) after the shunt surgery among 18 patients with iNPH (C).

Figure 7 and Table 4 show the changes in time and 95%CE volume for the 3D acceleration on the best performances in iTUG among 28 patients with tap-positive iNPH and 8 patients with tap-positive at the tap test and those among 18 patients with definite iNPH before and after the shunt surgery. Among 28 patients with tap-positive iNPH, the mean time on iTUG gradually shortened after the tap test from the first day to the fourth day, whereas the mean volume on 95%CE and the mean iTUG score were gradually increased (Table 4). Conversely, the mean iTUG score among 8 patients with tap-negative was gradually decreased after the tap test. After the shunt surgery among18 patients with definite iNPH, the mean time on iTUG was shortened by >5 s, the mean 95%CE volume was increased by >80 m^3^/s^6^ and the mean iTUG score was increased by >20 points. After the tap test, 18 tap-positive iNPH patients with an initial iTUG time of ≥13.5 s improved their iTUG time rather than increased 95%CE volumes, whereas 10 tap-positive iNPH patients with <13.5 s increased 95%CE volumes rather than decreased iTUG time (Fig. 7A and C). Consequently, the mean difference of iTUG time at the tap test among 18 tap-positive iNPH patients with ≥13.5 s was significantly larger, whereas the mean difference of 95%CE volume was significantly smaller than those among 10 tap-positive iNPH patients with <13.5 s (Table 5). However, the mean differences of iTUG scores were the almost same between the two groups of <13.5 and ≥13.5 s on initial iTUG time. In contrast, one tap-negative patient with <13.5 s decreased a 95%CE volume after the tap test and the other 7 tap-negative patients had small amounts of changes in the time or 95%CE volume for the 3D acceleration on iTUG (Fig. 7B).

**Table 3:** Characteristics and results on iTUG in the inpatient for other reasons and day-care user.

	Inpatient for other reasons (29)	Day-care user (87)	*P*
Man	18	40	0.20
Age, years	74.3 ± 7.4	79.5 ± 7.0	<0.01
***First time of iTUG***			
Time, second	11.9 ± 4.3	12.5 ± 4.1	0.35
95%CE volume, m^3^/s^6^	210.0 ± 256.8	143.2 ± 139.2	0.31
iTUG score	72.5 ± 38.4	62.8 ± 25.1	0.41
***Second time of iTUG***			
Time, second	11.7 ± 3.5	10.9 ± 3.0	0.42
95%CE volume, m^3^/s^6^	176.1 ± 139.2	183.8 ± 150.6	0.87
iTUG score	69.2 ± 25.7	71.8 ± 25.3	0.60

Day care user; Participants who exercise at the rehabilitation facility in the day care. 95%CE; 95% confidence ellipsoid for the tracks of the chronological changes of 3D acceleration on instrumented 3-m timed up-and-go test (iTUG).

### Distribution of iTUG score

**Figure 8.**
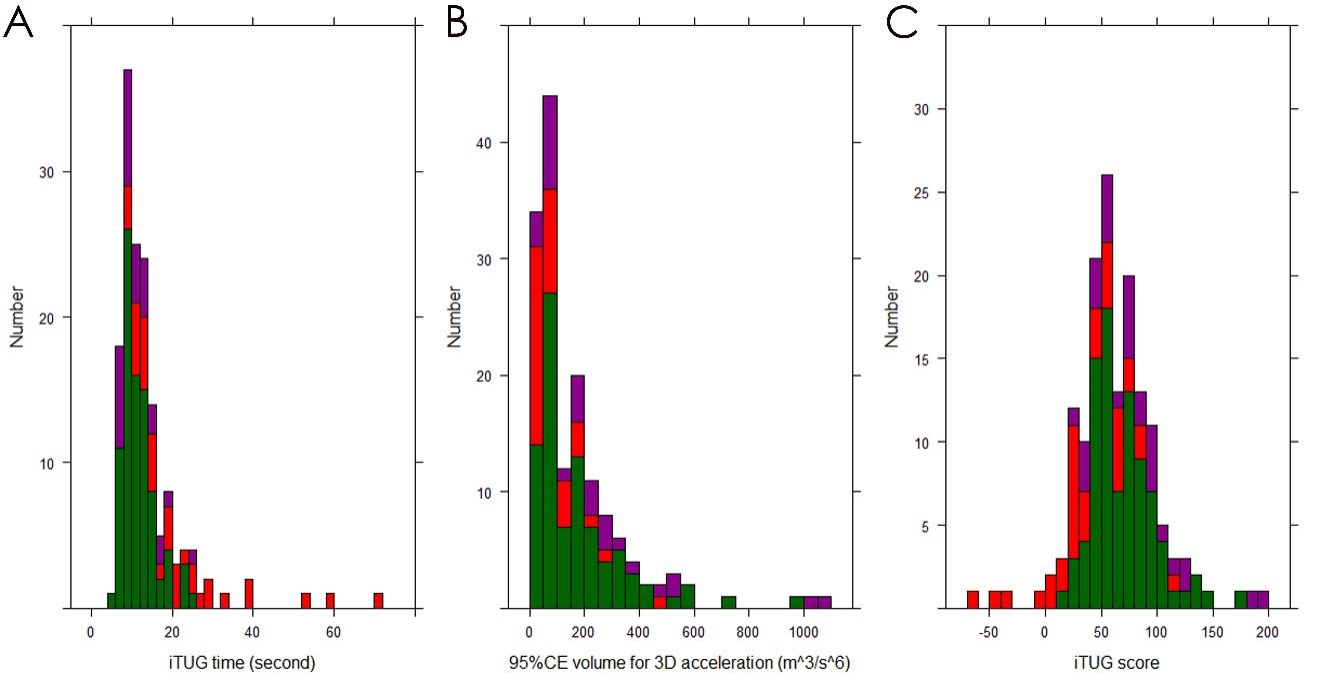
**Distributions of iTUG time, ellipsoid volume for 3D acceleration and iTUG score**. The y-axes show the numbers of patients with tap-positive iNPH before the tap test (red), patients hospitalized for other reasons (purple), and day-care users (green). Distributions of iTUG time (A) and ellipsoid volume for 3D acceleration (B) were biased distribution, but a distribution of iTUG score (C) approximately had a normal distribution.

The bar charts in Figure 8 represent the frequencies of time, 95%CE volume, and score on iTUG. The distributions of time and 95%CE volume on iTUG were biased toward low numerical values, whereas the distribution of the iTUG score approximately had a normal distribution.

### Reliability and repeatability

**Figure 9.**
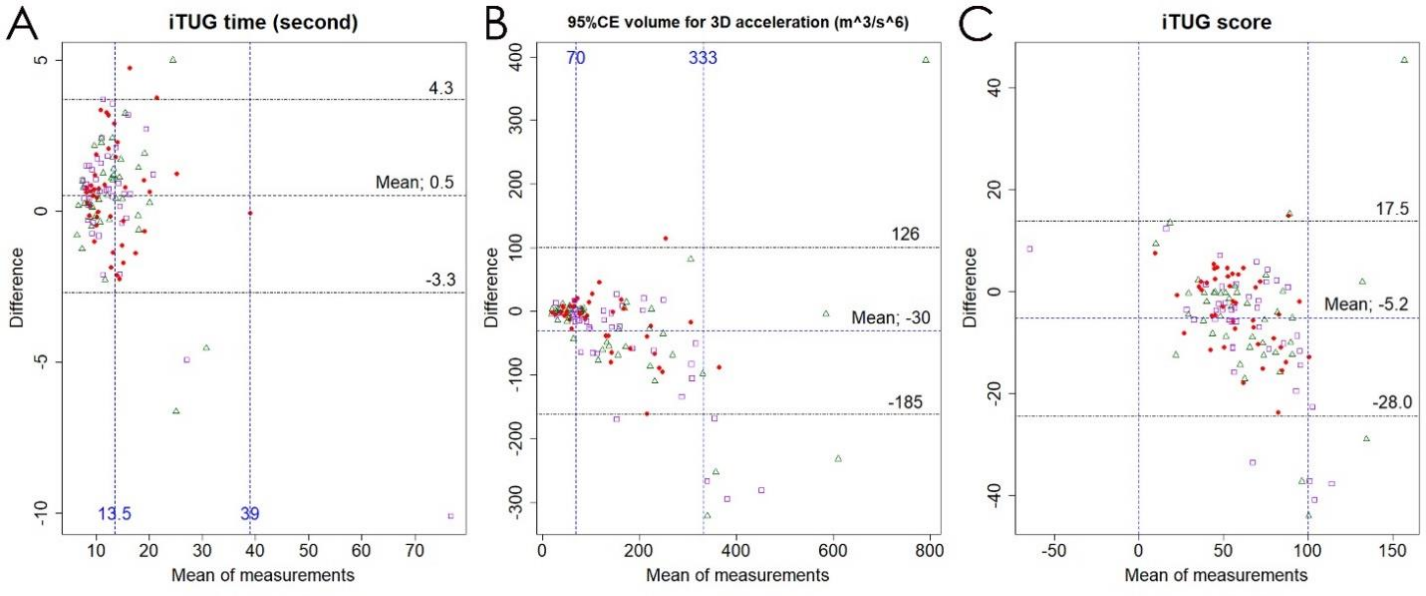
**Bland-Altman plots of iTUG time, ellipsoid volume for 3D acceleration and iTUG score**. The x-axes show the means of the first and the second measurements and the y-axes represent the differences between the two measurements on iTUG time (A), ellipsoid volume for 3D acceleration (B), and iTUG score (C). Red circles indicate patients with tap-positive iNPH before the tap test. Purple open squares indicate patients hospitalized for other reasons and did not undergo the tap test, and green open triangles indicate day-care users.

All three measurements on iTUG had significantly high ICCs for absolute test-retest reliability (p < 0.001): 0.97 (excellent reliability, 95% CI: 0.95-0.98) for the iTUG time, 0.80 (good reliability, 0.70-0.87) for the 95%CE volume, and 0.90 (excellent reliability, 0.82-0.94) for the iTUG score. Additionally, we assessed repeatability of iTUG using the Bland-Altman method (Fig. 9). The Bland-Altman plots revealed that there was a proportional bias in all three measurements on iTUG, and changes of ≤0.5 s in iTUG time, ≤30 m^3^/s^6^ in 95%CE volume and ≤5 points in iTUG score were regarded as a range of constant bias.

**Table 4:** Change of iTUG measurements at the best performance.

28 patients with tap-positive iNPH			
	Before tap	1 day after tap	4 days after tap
Time, s	23.8 ± 15.8	18.3 ± 10.2	15.1 ± 7.7
∆Time, s		-5.1 ± 10.1	-7.7 ± 9.8
95%CE volume, m^3^/s^6^	77.9 ± 65.2	101.8 ± 87.4	138.2 ± 114.2
∆95%CE volume, m^3^/s^6^		22.4 ± 49.8	52.7 ± 108.9
iTUG score	31.1 ± 35.4	45.4 ± 29.7	57.0 ± 28.4
∆ iTUG score		13.1 ± 19.3	22.0 ± 17.1
**8 patients with tap-negative**			
	Before tap	1 day after tap	4 days after tap
Time, s	16.4 ± 6.8	18.0 ± 10.0	16.4 ± 7.6
∆Time, s		1.6 ± 4.8	0.8 ± 2.8
95%CE volume, m^3^/s^6^	137.7 ± 149.0	120.9 ± 93.2	120.4 ± 81.8
∆95%CE volume, m^3^/s^6^		-16.8 ± 68.9	-31.5 ± 115.1
iTUG score	54.0 ± 32.1	49.1 ± 31.6	50.7 ± 26.0
∆ iTUG score		-4.9 ± 13.6	-7.0 ± 18.0
**18 patients with iNPH who underwent V-P shunt surgery**			
	Before shunt	After shunt	
Time, s	17.4 ± 5.0	11.8 ± 3.1	
∆Time, s		-5.7 ± 4.1	
95%CE volume, m^3^/s^6^	87.1 ± 53.3	163.7 ± 137.6	
∆95%CE volume, m^3^/s^6^		80.9 ± 110.2	
iTUG score	45.0 ± 15.5	67.1 ± 24.0	
∆ iTUG score		21.6 ± 16.2	

∆, difference before and after the tap test or shunt surgery. 95%CE; 95% confidence ellipsoid for the tracks of the chronological changes of 3D acceleration on instrumented 3-m timed up-and-go test (iTUG)

**Table 5:** Change of iTUG measurements in the groups with <13.5 and ≥13.5 seconds (s) on iTUG time.

Initial TUG time	<13.5 s	≥13.5 s	*P* value
Total number	10	18	
***Before tap test***			
iTUG time, second	11.7 ± 1.8	30.5 ± 16.2	<0.01
95%CE volume on iTUG, m^3^/s^6^	126.4 ± 74.8	50.9 ± 40.6	<0.01
iTUG score	62.4 ± 14.2	13.7 ± 31.4	<0.01
***1 day after tap test***			
iTUG time, s	10.3 ± 1.6	22.7 ± 10.3	<0.01
∆iTUG time, s	-1.4 ± 1.3	-7.2 ± 12.1	0.023
95%CE volume on iTUG, m^3^/s^6^	178.7 ± 97.9	59.2 ± 40.6	<0.01
∆95%CE volume on iTUG, m^3^/s^6^	60.9 ± 56.7	1.1 ± 29.8	<0.01
iTUG score	72.8 ± 16.8	30.2 ± 23.7	<0.01
∆ iTUG score	11.6 ± 10.0	14.0 ± 23.2	0.83
***4 days after tap test***			
iTUG time, s	9.5 ± 2.0	18.5 ± 7.9	<0.01
∆iTUG time, s	-2.2 ± 1.7	-10.9 ± 11.2	<0.01
95%CE volume on iTUG, m^3^/s^6^	227.5 ± 139.6	85.6 ± 48.2	<0.01
∆95%CE volume on iTUG, m^3^/s^6^	109.8 ± 148.2	19.2 ± 60.8	0.15
iTUG score	81.4 ± 22.0	42.7 ± 21.2	<0.01
∆ iTUG score	20.2 ± 22.4	23.1 ± 13.8	0.41

*P*; probability value of the group with <13.5 vs. ≥13.5s on iTUG time before the tap test ∆, difference before and after the tap test or shunt surgery. 95%CE; 95% confidence ellipsoid for the tracks of the chronological changes of 3D acceleration on instrumented 3-m timed up-and-go test (iTUG)

## DISCUSSION

In this study, we described a novel iTUG method using a free iPhone application that was able to assess not only the time but also the angular speed and acceleration in three axial directions by designing automatic analysis algorithms. After a systematic investigation, both the time and 95%CE volume for the tracks of the chronological changes of 3D acceleration on iTUG were found to play an important role in evaluating mobility. Moreover, the time and 95%CE volume for 3D acceleration on iTUG had a high accuracy and reliability. The time or gait velocity on TUG is reported to be insufficient to assess the effects of the tap test, including for diagnosing iNPH and selecting shunt candidates, especially in patients with mild gait disturbance [13, 15]. The cutoff times on TUG at a fast walking speed were reported to be 11-13.5 s for identifying individuals at an increased risk of falls [3, 8, 16-21]. Particularly, the most popular cutoff time on TUG at a fast pace for predicting falls is ≥13.5s [3, 8, 16, 18]. In this study, tap-positive iNPH patients with an initial TUG time of ≥13.5 s showed improved TUG time after the tap test, whereas those with a TUG time of <13.5 s did not demonstrate reduced time but showed increased ellipsoid volume on iTUG. This result confirms that the TUG time is a reliable measure for evaluating gait disturbance only at ≥13.5 s, and mild gait disturbance with a TUG time of <13.5 s should be evaluated with a focus on the 95%CE volume for 3D acceleration measured using iTUG rather than based on the TUG time. Many patients with iNPH whose TUG time is < 13.5 s have various gait disturbances and a history of falls. The reduced stride length (i.e. senile gait), diminished step height (shuffling), broad based gait, unsteady gait, antepulsion, and magnetic gait are known to be typical features of gait disturbance in iNPH [22, 23]. The reduced stride length might be mainly due to reduction of forward and vertical upward acceleration generated by kicking out with a toe. Reduction of vertical upward acceleration may be related with the diminished step height. Reduction of horizontal acceleration may cause the broad-based gait. Reduction of backward acceleration may cause the antepulsion. Further research is required to ascertain the relationship between the disturbed gait pattern in iNPH and characteristics of 3D acceleration during the iTUG.

The ideal gait assessment for clinical practice and research requires not only quantitative ability and repeatability but also simplicity and applicability. Therefore, we additionally evaluated iTUG in the other two heterogeneous groups, healthy elderlies and aged patients with various diseases. On the basis of the distribution and relationship between the time and 95%CE volume for the 3D acceleration on iTUG in the three groups, we newly created the iTUG score, which is reliable, valid, and feasible, and may be considered a new universal measure for evaluating gait disturbance. Furthermore, the changes in iTUG score adequately represent the changes in gait after the tap test or shunt surgery. For example, <5 improvement on iTUG score after the tap test is considered less improvement, ≥5 to <10 is slightly improvement, ≥10 to <20 is sufficient improvement, and ≥20 is excellent improvement. Previously reported instrumentations for evaluating gait disturbance and changes in patients with iNPH were too expensive and complicated for universal clinical use [15, 23-27]. In comparison with these methods, our iTUG score can be easily measured without the need for considerable amounts of time and money. Therefore, it is suitable for multicenter collaborative studies assessing gait disturbance.

This application automatically stopped in a person with severe gait disturbance, e. g. ≥40 s on TUG time or severe magnetic gait. In this study, 14% of patients for the tap test and 22% of patients with definite iNPH before shunt surgery could not accomplish iTUG. To address the issue, we would like to propose that an iTUG score should be applied an approximate value calculating as ‘67 - 1.9 × (time on TUG)’ at the application error, because a 95%CE volume for the 3D acceleration on iTUG in a person with severe gait disturbance estimates 25 m^3^/s^6^.

We conclude that the iTUG data measured using the free iPhone application have high reproducibility. For patients with mild gait disturbance, 3D acceleration, which mean the force to move, are more important for gait assessment than the simple time on TUG. The novel iTUG score, consisting of time and 95%CE volume for the 3D acceleration on iTUG, is a universally applicable method for the quantitative assessment of gait disturbance. Based on the results in this study, we made a new iPhone application “Hacaro - iTUG” (Digital Standard Co., Ltd., Osaka, Japan) which can be freely downloaded from the Apple store (https://itunes.apple.com/us/app/hacaro-itug/id1367832791?l=ja&ls=1&mt=8) and automatically calculate the iTUG score in concurrent with the time and 95%CE volume for the 3D acceleration on iTUG. We recommend the iTUG score as a next-generation international assessment measure for evaluating gait disturbance.
